# Differences in Gut Microbiome Composition and Antibiotic Resistance Gene Distribution between Chinese and Pakistani University Students from a Common Peer Group

**DOI:** 10.3390/microorganisms9061152

**Published:** 2021-05-27

**Authors:** Tianshu Feng, Mian Gul Hilal, Yijie Wang, Rui Zhou, Qiaoling Yu, Jiapeng Qu, Huan Li

**Affiliations:** 1 School of Public Health, Lanzhou University, Lanzhou 730000, China; godkuangxiaotian@163.com (T.F.); mianmalam@gmail.com (M.G.H.); 13196349170@163.com (Y.W.); zhour18@lzu.edu.cn (R.Z.); yuqiaoling1231@163.com (Q.Y.); 2 Key Laboratory of Adaptation and Evolution of Plateau Biota, Northwest Institute of Plateau Biology, Chinese Academy of Sciences, Xining 810008, China; 3 Center for Grassland Microbiome, Lanzhou University, Lanzhou 730000, China

**Keywords:** gut microbiota, nationalities, stabilization, antibiotic resistance genes (ARGs), human health

## Abstract

Gut microbiomes play important functional roles in human health and are also affected by many factors. However, few studies concentrate on gut microbiomes under exercise intervention. Additionally, antibiotic resistance genes (ARGs) carried by gut microbiomes may constantly pose a threat to human health. Here, ARGs and microbiomes of Chinese and Pakistanis participants were investigated using 16S rRNA gene sequencing and high-throughput quantitative PCR techniques. The exercise had no impact on gut microbiomes in the 12 individuals investigated during the observation period, while the different distribution of gut microbiomes was found in distinct nationalities. Overall, the dominant microbial phyla in the participants’ gut were Bacteroidota, Firmicutes and Proteobacteria. Some genera such as *Prevotella* and *Dialister* were more abundant in Pakistani participants and some other genera such as *Bacteroides* and *Faecalibacterium* were more abundant in Chinese participants. The microbial diversity in Chinese was higher than that in Pakistanis. Furthermore, microbial community structures were also different between Chinese and Pakistanis. For ARGs, the distribution of all detected ARGs is not distinct at each time point. Among these ARGs, *floR* was distributed differently in Chinese and Pakistani participants, and some ARGs such as *tetQ* and *sul2* are positively correlated with several dominant microbiomes, particularly Bacteroidota and Firmicutes bacteria that did not fluctuate over time.

## 1. Introduction

The human gut is a nutrient-rich environment; consequently, more than 100 trillion microbiomes have colonized it. As a result, the human gut is regarded as an essential microbial habitat in our biosphere [1]. Furthermore, these microbiomes represent about 25-times more genes than the human genome. For this reason, gut microbiomes are known as the second genome of the human body [2,3,4]. As an important part of the human microbiome, gut microbiomes play a crucial role in human health [5,6]. Firstly, the gut microbiome significantly impacts the absorption and metabolism of nutrients such as carbohydrates, proteins, and short-chain fatty acids. For instance, gut microbiota could influence energy storage, and participate in the degradation of dietary carbohydrates and cellulose [7,8]. Furthermore, gut microbiomes are also closely linked to the immune system [9,10]. Additionally, gut microbiomes are critical for the central nervous system because they play a role in the formation of the blood–brain barrier, myelination and neurogenesis [11]. Moreover, gut microbial imbalance probably leads to metabolic diseases such as obesity [12]. In addition, variations in the gut microbiome can mediate the effects of environmental factors on the risk of colorectal cancer [13].

Although gut microbiomes perform many functions, they are also influenced by various factors. Host genes are critical for the gut microbiome and significantly impact the microbial composition of the human gut [14,15,16]. Our microbial phenotype is usually influenced by our genetic state [14]. Additionally, the environment also plays a significant role in the composition of the human gut microbiome [17,18]. Among them, diet is commonly distinguished as an important factor for gut microbiomes because it helps in the modulation of gut microbiomes [19,20]. Except for these well-known factors, some researchers had found that exercise can affect the composition and diversity of gut microbiomes [21,22]. However, even though there are many influencing factors for gut microbiomes, gut microbiomes usually keep relatively stable, which is critical for human health. Like natural ecosystems, the microbial community in our gastrointestinal tract is a ‘resilient’ system with dynamics, stability and resilience [23]. Some recent studies in athletes, such as rugby players, revealed that exercise affects gut microbiomes [24,25]. However, the intensity and mode of exercises between athletes undergoing routine training and peoples who do not often exercise are distinct usually. It is possible that some other exercises such as jogging can also be interventions to investigate the variations of gut microbiomes.

As one of the most successful forms of chemotherapy in the medical history, antibiotics have saved millions of lives [26]. However, due to the abuse of antibiotics in recent years, the effectiveness of antibiotics in treating infections has rapidly declined [27]. Meanwhile, it causes a sharp accumulation and spread of ARGs. ARGs can be found in pristine environments such as the Antarctic and Amazonas [28,29,30]. However, the overuse of antibiotics increases the prevalence of ARGs in human and animal microbiota [31]. Now, antibiotic resistance genes (ARGs) are widely found in the gut [32], and most ARGs induced in animals are excreted into the environment via manure [33,34,35]. Antibiotic resistance has emerged as a major global public health issue in recent years [36,37]. The World Health Organization has considered antibiotic resistance one of the most serious public health threats of the twenty-first century [38]. As a new type of environmental pollutants [29,39], ARGs usually transfer and persist in the environment and may have more adverse impacts on the environment than the antibiotics themselves [40]. Antibiotic resistance genes spread rapidly worldwide. In Pakistan, the appearance of pan-resistant bacterial isolates caused septicemia [41]. China also faces the same problems [42,43]. Many ARGs have been detected likewise in Europe and America [39,44]. ARGs are usually correlated with gut microbiomes [32,45], thus the variations of gut microbiomes perhaps lead to the changes of ARGs. If the gut microbiomes change during exercise, do ARGs change? Furthermore, the differences in gut microbiomes between distinct nationalities may result in different ARGs. These are problems that need to be investigated.

In this study, daily jogging was used as an exercise for one month. In Chinese and Pakistanis from the same university, high throughput sequencing is used to analyze the composition and diversity of gut microbiota and quantitative PCR (qPCR) is used to quantify ARGs. This research aims to address the following scientific problems: (1) Whether intervention (exercises) and nationality lead to variations of composition and diversity in gut microbiomes in our selected subjects. (2) Whether the ARGs are distinct could be caused by altered gut microbiomes resulting from their close relationships.

## 2. Materials and Methods

### 2.1. Sample Collection

In this study, exercise intervention was jogging after dinner, and each exercise began at 7 pm and lasted for 25–35 min approximately. Here, 12 healthy participants (six Chinese and six Pakistanis) without smoking or drinking, and taking antibiotics in the last three months from Lanzhou University (North latitude 36°2′47″, East longitude 103°51′54″, altitude 1520 m) were recruited in October 2018. Their age ranged from 21 to 31 years and the average age was 25.58 years old when their information was collected. The number and information were listed in Table S1. All participants lived on the same campus during the observed period. The accommodations and facilities provided by the school were similar between Chinese and Pakistanis participants. There was only one canteen on this campus, and the canteen only offered limited and fixed foods containing similar food ingredients such as potatoes, chicken, tomatoes, cauliflower, and cabbage. Both the Chinese and Pakistani participants usually eat in the canteen. The experiment began immediately after the subjects were chosen. Fecal samples were collected before exercise, on the 7th, 14th, 21th and 28th days of continuous exercise. The samples were collected from 7 am to 10 am. Each exercise lasted 25–35 min. After frozen in the portable refrigerator (−20 ℃) for a short time, all samples were stored at −40 ℃ medical refrigerator in our laboratory immediately. Information of volunteers including age, gender, weight, height, and nationality had been collected. Additionally, their diet was recorded through interviews and was listed in Table S2. All subjects signed informed consent forms.

### 2.2. DNA Extraction, PCR Amplification and MiSeq Sequencing

Total DNA was extracted from Pakistani and Chinese fecal samples using the Ezup genomic DNA extraction kit (Sangon Biotech, Shanghai, China). The concentration and purity of DNA were quantitatively measured using the Nanodrop 2000 Spectrophotometer. Then, the extracted DNA was diluted to 10 ng/μL for PCR amplification. Next, the universal primers 515F (5′-GTGYCAGCMGCCGCGGTA-3′) and 909R (5′-CCCCGYCAATTCMTTTRAGT-3′) were used for amplifying the V4–V5 hypervariable region of microbial 16S rRNA gene [46]. When synthesizing primers, a 515F primer with a 12-bp barcode was inserted at the front end to distinguish each sample during the sequencing analysis [46].

PCR amplification was implemented using 25 μL reaction mix containing 1 × PCR buffer, 1.5 mM MgCl_2_, each deoxynucleoside triphosphate at 0.2 mM, each primer at 1.0 μM and 0.25 U of Ex Taq (TaKaRa, Dalian, China) and 10 ng genomic DNA. The next is thermal cycling. The first step was initial denaturation at 94 °C for 3 min, followed by 30 cycles of 40 s at 94 °C, 60 s at 56 °C, 60 s at 72 °C, and finally 10 min at 72 °C. After PCR amplification, the two PCR products were mixed and electrophoresis was performed using 1.2% agarose gel [47].

After completing PCR amplification, the band was excised and purified (~400 bp) with the SanPrep DNA Gel Extraction Kit (Sangon Biotech, Shanghai, China) and quantified with the Nanodrop 2000 Spectrophotometer. All samples were pooled together with an equal molar amount from each sample, and then, prepared samples for sequencing using the TruSeq DNA kit (vendor: Illumina; order number: FC-121–3003) according to the instruction of the manufacturer. The purified library was diluted, denatured, re-diluted, mixed with PhiX (equal to 30% of final DNA amount) based on the Illumina library preparation protocols and applied to an Illumina Miseq platform for sequencing (Reagent Kit V2) at the Environmental Genome Platform of Lanzhou University.

### 2.3. Bioinformatics Analysis

The QIIME Pipeline-Version 1.7.0 (http://qiime.org/tutorials/tutorial.html, accessed on 12 January 2019) was used to process the raw sequence data [48]. All sequences were trimmed and assigned to individual samples based on their barcodes. The FLASH-1.2.8 software was used to combine the overlapping paired-end reads [49]. The combined sequences with high quality (reads length >300 bp, without ambiguous base “N”, and average base quality score >30) were prepared for subsequent analysis. Due to possible contamination of chloroplast sequences in PCR amplification, we applied the Metaxa2 software tool [50] to remove chloroplast sequences from our large sequencing datasets for eliminating the effect that Chloroplast sequence is contaminated during the PCR amplification [51]. Then, we implemented the UCHIME algorithm to check and remove chimera of aligned 16S rRNA gene sequences [52]. All reads with a similarity higher than 97% were clustered into operational taxonomic units (OTUs) using CD-HIT [53]. Representative OTUs were picked out and identified by the Ribosomal Database Project classifier, then, singleton OTUs and non-bacterial OTUs were filtered out. Each sample was rarefied to the same number of reads (14246 sequences) to compare samples with different sequencing depths. We visualized stacked histogram at microbial phyla and genus level by Originlab 2017 (Originlab, Northampton, MA, USA) for describing the community composition of gut microbiomes. Next, the descriptions of microbiota distributed differently in Chinese and Pakistanis were presented by heatmap at phylum, genus and OTU level using “R” (“heatmap” package). The alpha diversity of Chinese and Pakistani participants was compared using a set of indices that included observed species, Shannon diversity, and the Simpson index. The microbial community was described using unweighted and weighted UniFrac distance matrices. Dissimilarities in Chinese and Pakistani gut microbial communities were calculated using Bray–Curtis distance matrices at each time point, and similarities are equal to “1-dissimilarity”. Internal similarities of gut microbial communities of participants from the same country were compared at each time point.

The original sequence data are available at the European Nucleotide Archive by accession number PRJEB41489 (http://www.ebi.ac.uk/ena/data/view/PRJEB41489, accessed on 23 November 2020).

### 2.4. Quantification of ARGs

After DNA extraction, we performed High-throughput quantitative PCR (HT-qPCR) of ARGs via the WaferGen SmartChip Real-Time PCR System in Hong Kong Microanaly Gene Technologies CO., Limited. Then we placed samples on the SmartChip Multisample Nanodispenser (MSND) using a 48 (assays) × 108 (samples) array. These genes included 11 target ARGs (*cfr*, *cmlA1-01*, *floR*, *qnrA*, *sul1*, *sul2*, *tetA-01*, *tetG-01*, *tetM-01*, *tetQ*, *vanA*). These ARGs were thought to confer resistance to the most commonly used antibiotics [54]. Primers used to amplify each gene are listed in Table S3 [55]. In addition, those ARGs appearing in at least one participant were listed in Table S4. Each 100 nL reaction mixture contained 50 nL 2× SYBR^®^ Premix Ex Taq™, 500 nM of the forward primers, 500 nM of the reverse primers, 1 nL of 0.1 μg /μL bovine serum albumin (BSA), and 19 nL of ddH_2_O, and 20 nL of a 3 ng/μL template DNA (Anhui Microanaly Gene Technologies company; Anhui, China). Amplification was proceeded in triplicate, and each primer had a non-template control. The thermal regimen included an initial denaturation at 95 °C for 10 min, followed by 40 cycles of denaturation at 95 °C for 30 s and annealing at 60 °C for 30 s, finally with a melting curve analysis was auto-generated by the program [56].

We used the SmartChip qPCR software (version 2.7.0.1; Anhui Microanaly Gene Technologies company; Anhui, China) to analyze the results of HT-qPCR. Wells with a multiple melting peaks or amplification efficiencies outside the range of 1.8–2.2 could not be analyzed. A limit was set on the number of cycles necessary to observe a significant fluorescence signal (threshold cycle, Ct). These samples were determined to be positive if they showed Ct < 31 cycles and more than two replicates amplification. The method for computing gene copy number is based on an equation described in a previous study [56].

### 2.5. Statistical Analysis

The Mann–Whitney U test, based on SPSS 21.0 (IBM, Armonk, NY, USA), was used to compare the distribution of gut microbiomes in Chinese and Pakistani, a series of alpha diversity indices, the similarity of gut microbiota, and the relative abundance of ARGs were compared. Permutational multivariate analysis of variance (PERMANOVA) and the Mantel test were used to confirm the impact of factors on gut microbiota using the R “Vegan” package based on unweighted and weighted UniFrac distance matrices. The difference in core microbiome had been analyzed using ‘linear discriminant analysis effect size’ (LEfSe) at https://huttenhower.sph.harvard.edu/galaxy/, accessed on 10 July 2020. Spearman’s correlation coefficients of relative abundance between gut microbiomes and ARGs were calculated by SPSS 21.0.

## 3. Results

### 3.1. Exercise Is Not Influencing the Gut Microbiomes

**Figure 1:**
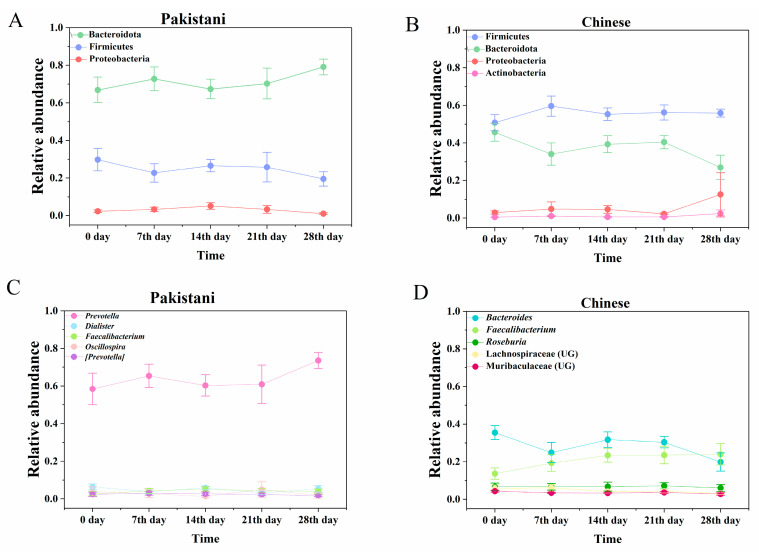
The same letters meant no statistical difference (*p* > 0.05) and different letters meant statistical difference (*p* < 0.05). Some dominant phyla (**A**,**B**) and genera (**C**,**D**) were compared in all time points based on the relative abundance via one-way ANOVA. Neither phyla nor genera were different among these 5 time points (*p* > 0.05).

According to PERMANOVA and Mantel test, daily exercises did not affect gut microbiomes, whereas different nationalities showed the differences in gut microbiomes of Chinese and Pakistanis (Table 1). In addition, body mass index (BMI; BMI = weight (kg)/height (m)^2^) also affected gut microbiomes, and average Pakistani BMI (24.32) was higher than that in China (20.20) (F = 126.187, *p* < 0.05) (Table 1). Similarities of gut microbiomes were the same at five time points (F = 1.656, *p* > 0.05) in Pakistanis, and similarities of gut microbiomes in Chinese were consistent at the first four time points whereas changed at 28th day (F = 5.940, *p* < 0.001, Figure S1A,B). Additionally, gut microbial diversity did not present a significant difference at each time point for both Pakistanis and Chinese (Figure S1C,D). Moreover, we selected dominant microbial phyla (relative abundance >1%) and genera (top 5) in Chinese and Pakistanis respectively to investigate their temporal variations in a month. We found that these selected dominant microbial phyla or genera did not vary significantly during exercise intervention in Chinese and Pakistanis individually (Figure 1A–D).

### 3.2. Gut Microbiomes Were Distributed Differently in Chinese and Pakistani Participants

Among the variables studied, nationality was found to be closely related to the gut microbiome. Following that, we described the characteristics of participants’ gut microbiomes based on their nationalities.

**Figure 2:**
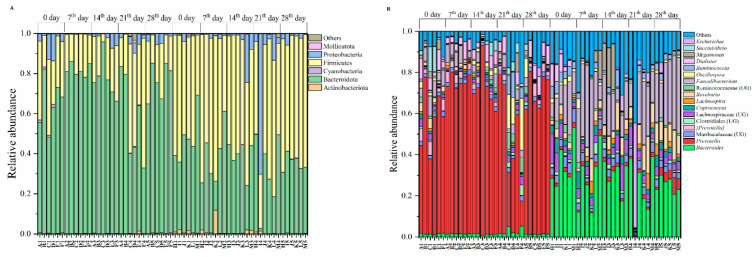
The composition of dominant gut microbiota in Pakistanis and Chinese at the phyla level (**A**) and genus level (**B**). The identifiers of 6 Pakistani participants are A–F, and the identifiers of 6 Chinese participants are H–M, and 1–6 represented different time point, 0 day, 7th day, 14th day, 21th day and 28th day. For example, A3 represented the Pakistanis participant marked A in 14th day.

**Figure 3:**
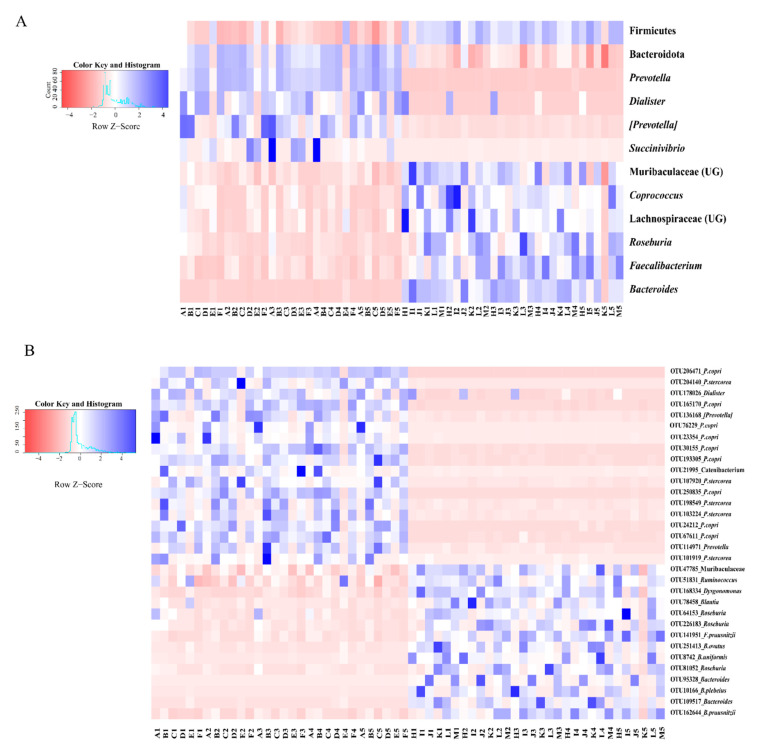
The comparison of dominant gut microbiota between Pakistani and Chinese participants at the phyla level, genus level (**A**), and OTU level (**B**). We picked phyla with average relative abundance >0.1% and genera with average relative abundance >1% individually. At OTU level, relative abundance ranked top 10 were selected. Only microbiomes distributed significantly different using Mann–Whitney U test between Chinese and Pakistanis were presented (*p* < 0.05).

**Figure 4:**
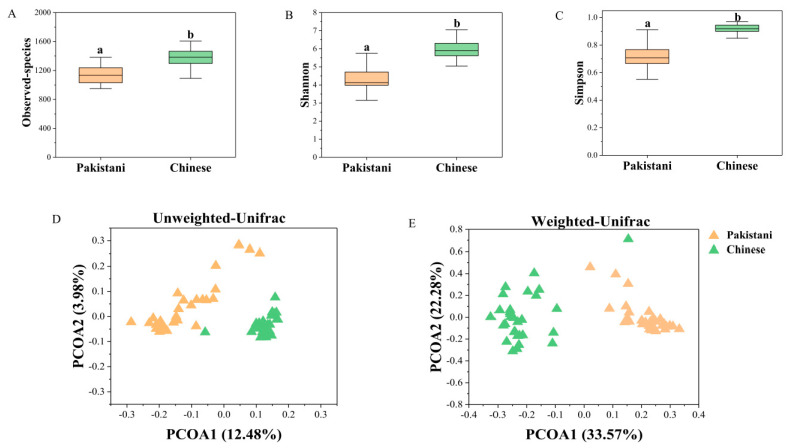
The comparison of alpha diversity based on observed-species (**A**), Shannon diversity (**B**), Simpson (**C**), and microbial community (**D**,**E**) between Chinese and Pakistani. The alpha diversity was compared by the Mann–Whitney *U* test. The mark of (a) and (b) indicated a significant difference between two groups (*p* < 0.05). Community structure with obvious separations were compared by PCOA based on unweighted uniFrac distance (**D**) and weighted uniFrac distance (**E**).

For the composition of gut microbiomes, at the phylum level (Figure 2A), the dominant microbiomes (Average relative abundance >0.1%) were the Bacteroidota (54.30%), Firmicutes (40.40%), Proteobacteria (4.21%), Actinobacteriota (0.67%), Mollicutota (0.11%) and Cyanobacteria (0.11%). At genus level (Figure 2B), the dominant microbiomes (Average relative abundance >1%) included *Prevotella* (32.94%), *Bacteroides* (15.11%), *Faecalibacterium* (12.48%), *Roseburia* (3.95%), etc. Moreover, there were many differences in the distribution of gut microbiota (Figure 3). At the microbial phylum level (Figure 3A), the Bacteroidota was more abundant in Pakistanis, whereas the Firmicutes was more abundant in Chinese. At the microbial genus level (Figure 3A), the most abundant genus in Pakistanis was *Prevotella*, but the most dominant genus in Chinese was *Bacteroides*. At the OTU level (Figure 3B), OTUs enriched in the Pakistanis were mainly included in *P*. *copri* and *P. stercorea*, while OTUs enriched in the Chinese were included in *Roseburia*, *Bacteroides* and *F. prausnitzii* mostly. However, many gut microbiomes were shared by Pakistani and Chinese people. At the genus level (Figure S2A), 161 bacterial genera (66.8%) were shared. At the OTU level (Figure S2B), 4491 common OTUs accounted for 46.4% proportion. The diversity and community structure of gut microbiomes are also distinct in different nationalities. In this study, the alpha diversity of gut microbiomes in the Chinese participants was higher than that in the Pakistani participants (*p* < 0.05; Figure 4A–C). Community structures of gut microbiota were also different between people with different nationalities (Table 1). There was an obvious difference in the gut microbiomes between Chinese and Pakistanis (Figure 4D,E).

**Figure 5:**
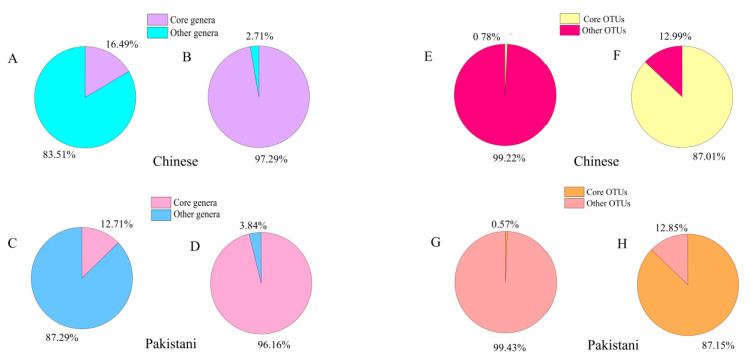
The proportion of specie-numbers and the relative abundance of core microbiome in Pakistani and Chinese at genus level (**A**–**D**) and OTU level (**E**–**H**).

Next, we described the core microbiome in Chinese and Pakistani participants. A core microbiome usually consist of members shared by two or more microbial assemblages [57]. In this study, core microbiomes were defined as microbiomes found in all samples at the genus level and in 80% of samples at the OTU level. At the microbial genus level (Figure 5), the core genera in Chinese accounted for 16.49%, but their relative abundance was 97.29%. Similarly, the core genera in Pakistani only accounted for 12.71%, while their relative abundance was 96.16%. Results at the OTU level were paralleled to the genus level (Figure 5). The number of these microbiomes accounted for a small proportion, whereas the relative abundance of these microbiomes was extremely high. Then, according to the average relative abundance, the top ten core microbiomes at the genus level were identified in Chinese and Pakistanis respectively (Figure S3). The core microbiome with the highest relative abundance was the *Bacteroides* in Chinese and the most abundant core microbiome was the *Prevotella* in Pakistanis (Figure S3A,B). The distributions of core microbiomes were distinct between Chinese and Pakistanis (Figure S3C).

### 3.3. Distributions of Antibiotic Resistance Genes (ARGs) in Participants and Relationships between ARGs and Gut Microbiota

**Figure 6:**
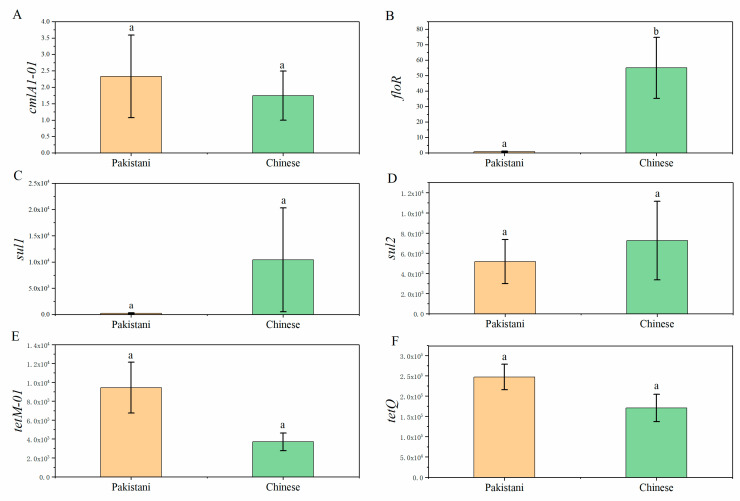
The comparison of ARGs using Mann–Whitney U test between Chinese and Pakistani. The mark of (a) and (b) indicated a significant difference between two groups (*p* < 0.05). (**A**): Comparison of *cmlA1-01*; (**B**): Comparison of *floR;* (**C**): Comparison of *sul1*; (**D**): Comparison of *sul2*; (**E**): Comparison of *tetM-01*; (**F**): Comparison of *tetQ*.

**Figure 7:**
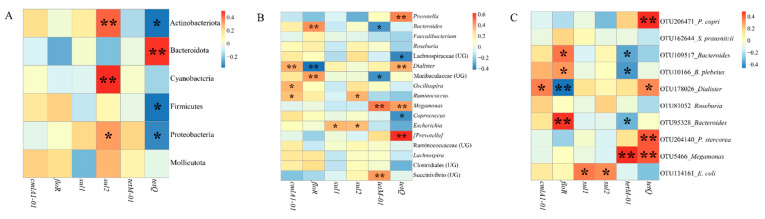
The correlations between gut microbiomes and ARGs at phylum (**A**), genus (**B**) and OTU level (**C**). * *p* < 0.05; ** *p* < 0.01.

In our research, 6 ARGs including *cmlA1-01*, *floR*, *sul1*, *sul2*, *tetM-01* and *tetQ* were detected in at least one participant at each time point, and *sul2*, *tetM-01* and *tetQ* appeared in all participants (Table S4). Only *floR* was different between Chinese and Pakistanis (Figure 6). Following that, Spearman’s correlation coefficient was used to describe the relationships between dominant gut microbiomes and ARGs (Figure 7) at the phylum level (Average relative abundance >0.1%), genus level (Average relative abundance >1%), and OTU level (Top 10 of average relative abundance). Some dominant gut microbiomes presented positive correlations with ARGs. For example, at the phylum level, *sul1* was positively correlated with the Cyanobacteria, Actinobacteriota and Proteobacteria, and *tetQ* was positively correlated with the Bacteroidota (Figure 7A). Several dominant genera such as *Dialister* and *Prevotella* presented positive correlations with ARGs such as *tetQ* (Figure 7B).

## 4. Discussion

### 4.1. Gut Microbiomes Remain Stable under the Intervention of Exercises during the Investigation Period

Recently, some studies have illustrated that physical activity could affect the gut microbiota. Gut microbiomes between athletes and sedentary controls are different because of distinct exercise intensity, and prolonged, intense exercise possibly positively influences gut microbial diversity and increases the relative abundance of some bacterial species usually [24,58,59]. However, these studies mainly focus on athletes who do intense exercise usually. In our study, exercise cannot alter the composition and diversity of gut microbiomes in selected Chinese and Pakistanis, which probably attributes to short exercise time (each exercise lasts for 25–35 min) and observation period (one month). Another study also confirmed that short-term high-intensity interval training exercise did not alter bacterial community structure [60]. This phenomenon is possibly interpreted as ‘temporal stability’ of gut microbiota [23,61].Participants in this study are all adults, and gut microbiomes are usually relatively stable [62,63].

### 4.2. Different Nationalities Cause Variations of Gut Microbiomes

In different nationalities, gut microbiomes differ significantly in composition, diversity, microbial communities, and core microbiomes. Many studies have found that people of different nationalities have different gut microbiomes. For example, gut microbiomes of Malawians, Amerindians and Americans differ [64]. Similarly, a study conducted in France, Germany, Italy, and Sweden concludes that nationalities are also shape the gut microbiomes [65]. Furthermore, there was no significantly difference in the diversity of human gut microbiota and community structure across different regions of the same country, but bio-diversity and community structure are significantly different across distinct counties [66]. Differences in gut microbiomes between Chinese and Pakistanis are observed in the current study. Perhaps these differences are due to genetic diversity, varying long-term living environments, and diet.

Firstly, genetic diversity is usually an essential factor for gut microbiomes. According to one study, the genetics of host has an impact on alpha diversity, beta diversity, and genome sequence similarity, which is positively correlated with microbiome similarity [67]. For example, compared to dizygotic twins, genetics are more similar in identical twins, consequently, the composition of gut microbiomes in identical twins is more analogous [14]. Furthermore, the environmental factors are likely to shape gut microbiome composition, and differences in gut microbiomes are likely to be attributed to different environments [18,68]. An investigation observed that individuals sharing a household had significant similarity in gut microbiomes, whereas there was no significant microbiome similarity among relatives who did not share a household in the past [18]. Moreover, one study revealed that dogs and people living in the same household had shared microbiomes [69]. Even if all participants spend two years in the same environment when samples were collected, they may have differences in gut microbiomes when their native environment was different. Participants from Pakistan only lived in this environment for a few years, but they spend many years (22–28 years) in their native environment. Furthermore, diet is a major factor influencing gut microbiomes, and long-term dietary patterns can modulate gut microbiome composition [70]. The eating habits and preferred foods are different in Chinese and Pakistanis, for example, the Pakistanis feed mainly on chicken, mutton and beef, however, Chinese people tend to take pork as their main meat food, additionally, the main cooking method is stewing in Pakistan whereas cooking methods are diverse in China such as frying, boiling and steaming. These factors of diet are usually related to gut microbiomes [71,72].

### 4.3. Some Dominant Gut Microbiomes Are Shared in People from Different Counties

Those shared gut microbiomes in Chinese and Pakistanis can be found in almost every individual and account for a significant proportion. At the microbial phylum level, Bacteroidota and Firmicutes are the most abundant microbial phyla in Chinese and Pakistani respectively. Generally, the healthy gut microbiomes are dominated by the phyla Firmicutes and Bacteroidota [73,74]. Both the Firmicutes and Bacteroidota play important roles in the human body such as metabolic and immunological functions, and the most abundant gut microbiomes usually belong to these phyla [75]. In our study, the phyla Bacteroidota dominates gut microbiomes in Pakistanis, whereas the phyla Firmicutes dominates gut microbiomes in Chinese. Many enzymes for hydrolyzing complex carbohydrates can be encoded by Firmicutes and Bacteroidota [6], which is important in our daily life. Bacteroidota is a highly efficient carbohydrate metabolizer with greater carbohydrate hydrolytic enzyme diversity and genomic content [76,77]. Compared with Bacteroidota, the Firmicutes contain fewer carbohydrate hydrolytic enzymes [78]. In addition, Firmicutes and Bacteroidota can regulate the immune system by inducing cytokines [79]. At the genus level, *Prevotella*, *Bacteroides* and *Faecalibacterium* are the most abundant microbial genera. These microbiomes are often used as indicators of healthy gut microbiota [80]. Some functions of *Prevotella* and *Bacteroides* are beneficial for humans. For example, the *Bacteroides* species are saccharolytic bacteria and can degrade dietary polysaccharides and glycans [81]. The *Prevotella* can improve glucose metabolism [82]. However, the distributions of *Prevotella* and *Bacteroides* differ Chinese and Pakistani participants. The different distributions of these two microbial genera are related to diet. A high intake of fat and protein usually leads to an increase in *Bacteroides*, but a high intake of fiber leads to an increase in *Prevotella* [82,83]. Perhaps the difference is due to the different diets of Chinese and Pakistanis. Based on *Prevotella* and *Bacteroides*, the term of ‘enterotypes’ was introduced in some studies. Typically, there are two types of communities, some communities are dominated by *Prevotella*, and others are dominated by *Bacteroides* [83,84]. Furthermore, the differences in the distribution of *Prevotella* and *Bacteroides* may be due to their antagonistic relationship [83,85]. Both *Prevotella* and *Bacteroides* belong to Bacteroidota, and when species of both microbial genera present in the gut, only one of them can dominate [85]. The core microbiome is essential to the ecology of the microbial community [57]. Core microbiomes have the same characteristics for both Chinese and Pakistani participants: the number of core microbiomes is less, but their relative abundance is enormous. However, the distribution of the core microbiome is distinct. Only five genera, *Bacteroides*, *Prevotella*, *Faecalibacterium*, Muribaculaceae (UG) and Lachnospiraceae (UG), are shared by Chinese and Pakistanis in the top ten core genera. Among these core genera, *Bacteroides* and *Prevotella* differ significantly between participants of different nationalities, and one study also found that the most variations in the core microbiome were *Bacteroides* and *Prevotella* [86].

### 4.4. Some Dominant Gut Microbiomes Have the Potential as Reservoirs of Antibiotic Resistance Genes (ARGs)

Horizontal gene transfer (HGT) allows human gut microbiomes to acquire ARGs from other bacteria in the external environment [32]. As a result, the human gut microbiome typically contains a diverse range of ARGs [32]. The gut microbiome is thought to be an ARGs reservoir [45,87]. It has been established that some microbiomes such as Proteobacteria may serve as a reservoir for ARGs such as *sul1*, *sul2*, *tetA*, *tetB*, *tetG*, *cml*, etc. [88]. Similarly, Actinobacteriota are the primary carriers of *sul2* [89]. In our study, Proteobacteria and Actinobacteriota have positive correlations with *sul2* in our study. Some dominant microbiomes, such as *Prevotella* and *Bacteroides*, are positively correlated with several ARGs at the genus level, such as *floR* and *tetQ*. However, these gut microbiomes with high relative abundance do not change over time in our study. Whether those dominant gut microbiomes that keep ARGs in the body for long term deserves more attention.

Finally, we admit that our study has some flaws due to the small number of participants and short observation periods. Perhaps short-term mild exercise is an interesting intervention for gut microbiomes; we need to recruit more participants and keep them for an extended time period in future studies with a more specific measurement of exercise intensity. Furthermore, we only consider a few factors (nationality and exercise) in this study, yet gut microbiomes are affected by many factors, therefore, we should include more factors in future. Moreover, gender probably influences gut microbiomes, but the gender ratio is not well balanced between the Chinese and Pakistani participants, which hampers our ability to draw general conclusions. Therefore, we will broaden the sampling range to ensure a balanced gender ratio.

## Figures and Tables

**Table 1:** PERMONOVA and Mantel test determining the influencing factors of gut microbial communities.

	Permanova				Mantel Test			
	Unweighted UniFrac		Weighted UniFrac		Unweighted UniFrac		Weighted UniFrac	
	R^2^	*p*	R^2^	*p*	r	*p*	r	*p*
Country	0.1023	**<0.001**	0.3120	**<0.001**	0.658	**<0.001**	0.583	**<0.001**
BMI	0.0731	**<0.001**	0.2280	**<0.001**	0.401	**<0.001**	0.418	**<0.001**
Exercise	0.0162	0.515	0.0116	0.645	−0.038	0.754	−0.059	0.839

(Bold font means the statistical significance. Unweighted UniFrac and Weighted Unifrac mean Permanova and Meantal test were done based Unweighted UniFrac and Weighted Unifrac distance matrices. Unweighted UniFrac only considers the presence or absence of species, while weighted UniFrac considers both the presence or absence of species and the species abundance.)

## Data Availability

The raw data of oral microbiota were deposited by accession number PRJEB41489 into the European Nucleotide Archive (http://www.ebi.ac.uk/ena/data/view/PRJEB41489 accessed on 31 March 2021). Other datasets presented in this article are available with requests directed to H.L., School of Public Health, Lanzhou University, Lanzhou China.

## Supplementary Materials

The following are available online at https://www.mdpi.com/article/10.3390/microorganisms9061152/s1, Figure S1: The changes of similarities and diversity of gut microbiomes during observation period in Chinese and Pakistani. The similarities and diversities were compared by one-way ANOVA in each time point for Chinese and Pakistani respectively (A–D), Figure S2: Shared gut microbiota and unique gut microbiota in Pakistani and Chinese at genus level (A) and OTU level (B), Figure S3: The rank of core gut microbiome in Pakistani participants (A) and Chinese participants (B) at genera level, and the different core genera in Chinese and Pakistani participants (C), Table S1: The information list of all participants, Table S2: Food composition of Chinese and Pakistani, Table S3: Primers for antibiotic resistant genes, Table S4: The list of Detected ARGs (copy number), these ARGs are detected in at least one participant at each time point. The identifiers of 6 Pakistani participants are A–F, and the identifiers of 6 Chinese participants are H–M, and 1–6 represented different time point, 0 day, 7th day, 14th day, 21th day and 28th day. For example, A3 meant the Pakistanis marked A in 14th day.
