# Supplementary material for: Differences in Gut Microbiome Composition and Antibiotic Resistance Gene Distribution between Chinese and Pakistani University Students from a Common Peer Group
Source: Microorganisms. 2021 May 27;9(6):1152. doi: 10.3390/microorganisms9061152 (PMC8229524; doi:10.3390/microorganisms9061152)
Supplement: Supplementary file 1 [file microorganisms-09-01152-s001.zip › microorganisms-1181846-supplementary.pdf]

Human Gut Microbiomes and Antibiotic Resistance Genes Differ in Selected Chinese and Pakistani

Tianshu Feng[1], Mian Gul Hilal[1], Yijie Wang[1], Rui Zhou[1], Qiaoling Yu[1], Jiapeng Qu[3]*, Huan Li[1, 2]*

1.Public health school of Lanzhou University, Lanzhou, 730000, Gansu

2. Center for Grassland Microbiome, Lanzhou University, Lanzhou, 730000, China

3. Key Laboratory of Adaptation and Evolution of Plateau Biota, Northwest Institute of Plateau Biology, Chinese Academy of Sciences, Xining, Qinghai, 810008, China.

Corresponding author, Huan Li, *E-mail lihuanzky@163.com (H.L.); Tel. +86-0931-8915008; Fax +86-0931-8915008. *E-mail jpqu@nwipb.cas.cn (J.Q.); Tel. +86-971-6143610; Fax +86-971-6143282.

Running title: Country rather than exercise affect gut microbiomes.

Table S1 The information list of all participants

Number	Gender	Height (cm)	Weight (kg)	BMI	Age (years)	Blood group	Nationality	Ethnic group
A	Male	173	74	24.9	30	B+	Pakistani	Punjabi
B	Male	172	64	21.6	24	O+	Pakistani	Punjabi
C	Male	165	66	24.4	29	B-	Pakistani	Punjabi
D	Male	176	78	25.2	31	AB+	Pakistani	Punjabi
E	Male	171	72	24.7	28	A+	Pakistani	Punjabi
F	Male	170	73	25.1	28	AB+	Pakistani	Punjabi
H	Female	160	54	21.1	22	AB+	Chinese	Han
I	Female	172	56	18.9	21	B+	Chinese	Han
J	Female	162	55	21	22	A+	Chinese	Han
K	Female	163	52	19.6	21	A+	Chinese	Han
L	Female	170	52	18	21	A+	Chinese	Han
M	Male	182	75	22.6	26	NA	Chinese	Han

Table S2 Food composition of Chinese and Pakistani

	staple food	Vegetables and fruits	Protein food	Non staple food
Pakistani	Bread, Rice, Potatoes, Bean Noodles,Bean, Cakes, Burger, Buiscuit	Cauliflower,Cucumber,Cabbage,Carrots, Apples, Bananas, Tomatoes, Pumpkin, Oranges, Vegetable Salad	Beaf, Eggs, Chiken, Fish,Tofu, Milk, Mutton	Pickle, Tea, Yogurt, Honey
Chinese	Porridge, Bread, Rice,Potatoes, Bean, Noodles, Sweet potatoes, Cakes, Hamburger, Corn	Cauliflower, Lettuce, Nori, Cabbage, Carrots, Tomatos, Radishes, Garlic bolt, Kelp,Apples, Oranges, Bananas	Pork, Shrimps, Fish, Eggs, Beaf, Milk, Soybean, Sausages, Chiken, Tofu	Pepper, Sauce, Yogurt, Dessert, Choclate,

Table S3 Primers for antibiotic resistant genes

Gene Name	Forward Primer	Reverse Primer
16S rRNA	GGGTTGCGCTCGTTGC	ATGGYTGTCGTCAGCTCGTG
cfr	GCAAAATTCAGAGCAAGTTACGAA	AAAATGACTCCCAACCTGCTTTAT
cmlA1-01	TAGGAAGCATCGGAACGTTGAT	CAGACCGAGCACGACTGTTG
floR	ATTGTCTTCACGGTGTCCGTTA	CCGCGATGTCGTCGAACT
qnrA	AGGATTTCTCACGCCAGGATT	CCGCTTTCAATGAAACTGCAA
sul1	CAGCGCTATGCGCTCAAG	ATCCCGCTGCGCTGAGT
sul2	TCATCTGCCAAACTCGTCGTTA	GTCAAAGAACGCCGCAATGT
tetA-01	GCTGTTTGTTCTGCCGGAAA	GGTTAAGTTCCTTGAACGCAAACT
tetG-01	TCAACCATTGCCGATTCGA	TGGCCCGGCAATCATG
tetM-01	CATCATAGACACGCCAGGACATAT	CGCCATCTTTTGCAGAAATCA
tetQ	CGCCTCAGAAGTAAGTTCATACACTAAG	TCGTTCATGCGGATATTATCAGAAT
vanA	AAAAGGCTCTGAAAACGCAGTTAT	CGGCCGTTATCTTGTAAAAACAT

Table S4 The list of Detected ARGs (copy number), these ARGs are detected in at least one participant at each time point. The identifiers of 6 Pakistani participants are A, B, C, D, E, F, and the identifiers of 6 Chinese participants are H, I, J, K, L, M, and 1, 2, 3, 4, 5, 6 represented different time point, 0 day, 7th day, 14th day, 21th day and 28th day. For example, A3 meant the Pakistanis marked A in 14th day.

	cmlA1-01	floR	sul1	sul2	tetM-01	tetQ
A1	6.647	1.227	0	19138.202	25320.900	403154.157
B1	0	0	610.363	1692.772	5928.264	704260.867
C1	18.034	4.171	0	1335.103	3317.949	34833.252
D1	0	0	507.364	279.332	7633.082	347565.736
A2	0	0	0	3045.888	4760.494	376845.211
B2	0	0	217.062	1011.512	2769.039	370413.665
C2	5.403	3.127	0	712.819	721.897	152254.101
D2	0	0	450.964	330.414	15250.286	138666.004
A3	0	0	31.971	41409.692	53517.399	193338.346
B3	0	0	42.475	679.190	1482.068	277582.447
C3	21.213	4.142	26.545	1357.222	1829.650	132149.645
D3	0	1.586	1944.979	272.902	19186.864	174448.002
A4	0	0	12.151	24375.695	29233.512	139819.260
B4	0	0	89.433	699.130	6330.281	350692.921
C4	0	2.190	3.453	1265.768	1746.803	205977.129
D4	0	0	202.154	260.743	1355.929	248556.429
A5	0	2.811	14.320	5816.088	10207.185	299289.858
B5	0	0	101.527	684.162	2750.813	281665.724
C5	0	0	9.179	529.894	698.334	133222.016
D5	0	0	137.420	26.838	4120.235	170398.591
H1	14.481	1.352	0	4227.251	705.646	242904.187
I1	0	157.717	0	9138.397	441.108	553505.576
J1	1.178	158.672	320.108	81.800	9768.458	253249.735
K1	0	0	80.284	10758.928	1161.323	93507.619
H2	4.256	0	1239.002	5709.368	2838.448	228332.115
I2	0	0	0	613.256	827.081	77159.073
J2	0	392.684	0	346.192	5139.442	634036.121
K2	0	0	0	786.625	1511.367	5779.842
H3	7.572	0	1884.551	3180.819	615.467	194854.751
I3	0	13.236	21.795	3448.260	533.836	113495.593
J3	0	66.614	5.761	21.342	2999.535	160613.853
K3	0	24.851	506.323	2012.301	1759.070	23645.636
H4	4.445	0	4106.398	12939.306	1274.304	140683.447
I4	0	0	10.258	409.027	3231.670	21948.0529
J4	0	100.305	100.305	748.175	13359.740	233575.883
K4	0	9.650	1412.313	8983.240	7746.473	105533.903
H5	3.685	0	1551.372	6028.552	858.960	183903.079
I5	0	0	6.836	439.508	8539.885	1878.495
J5	0	102.481	9.746	26.428	14926.260	209499.987
K5	0	71.079	217777.997	86811.248	1819.240	90.538

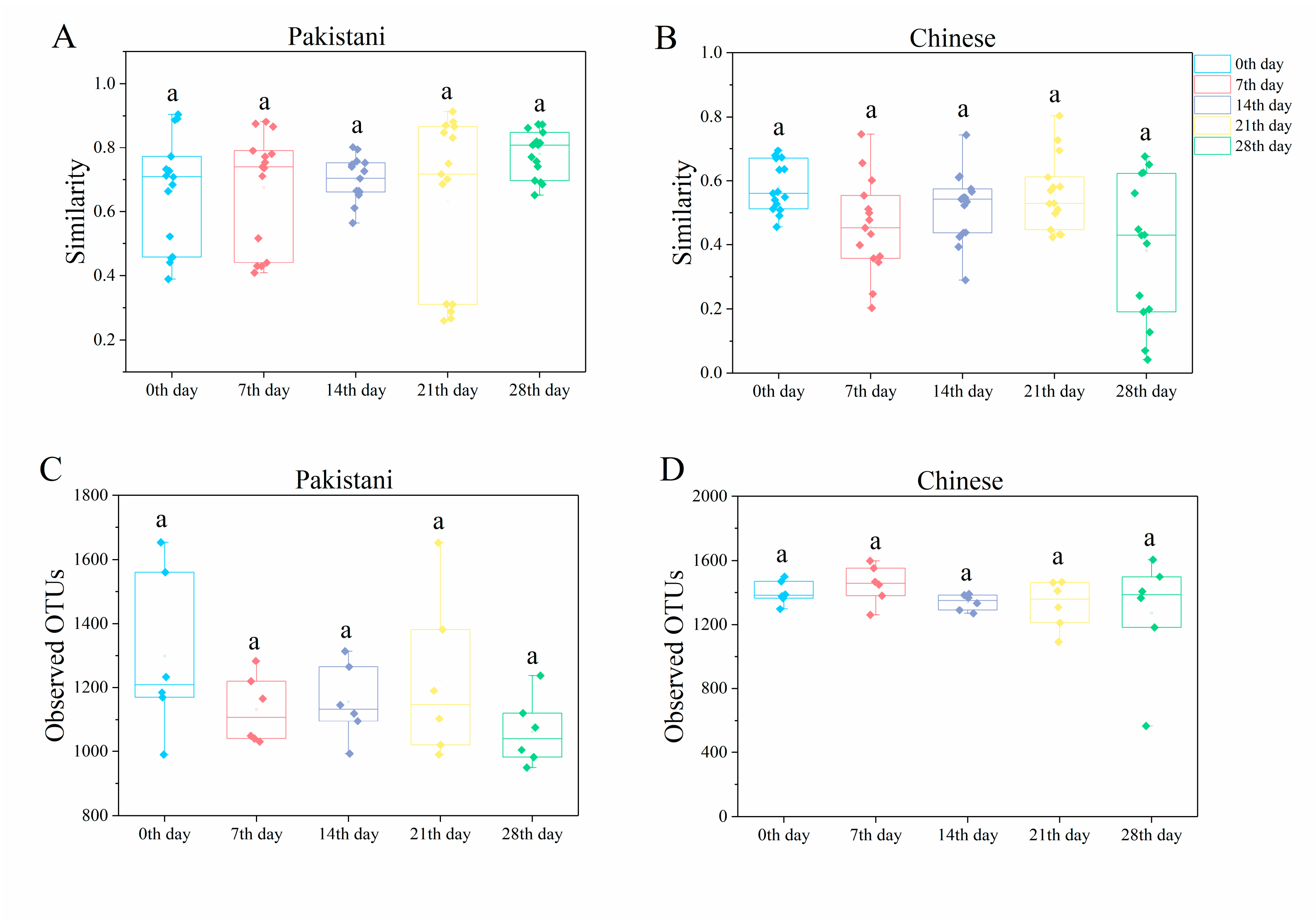

Figure S1 The changes of similarities and diversity of gut microbiomes during observation period in Chinese and Pakistani. The similarities and diversities were compared by one-way ANOVA in each time point for Chinese and Pakistani respectively (A-D).

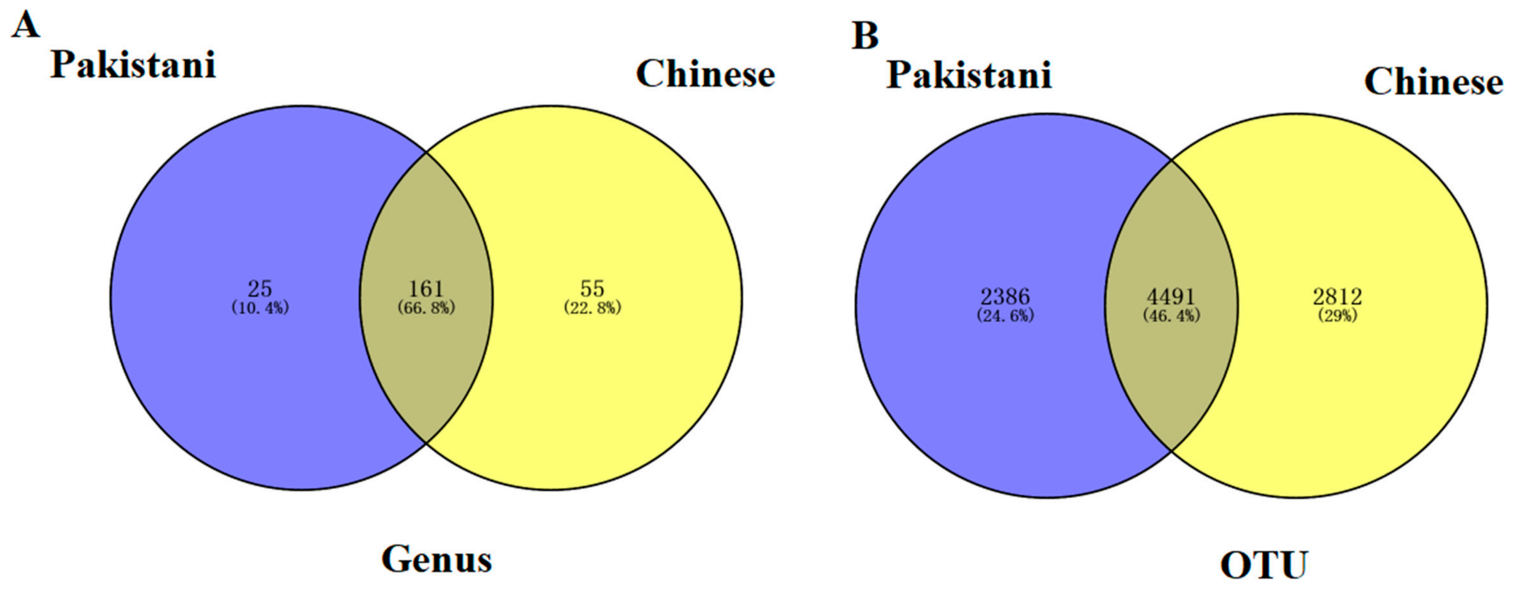

Figure S2 Shared gut microbiota and unique gut microbiota in Pakistani and Chinese at genus level (A) and OTU level (B).

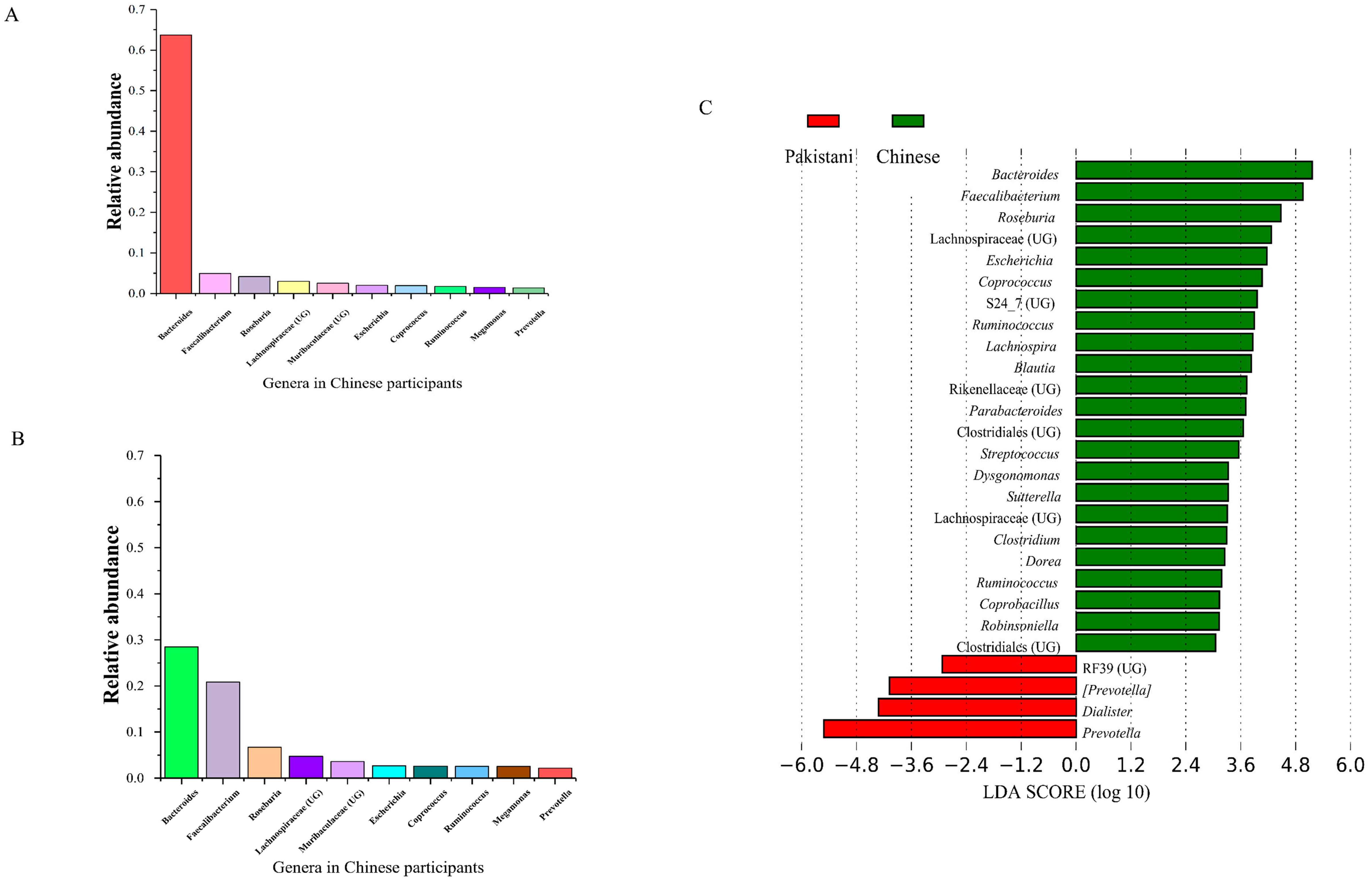

Figure S3 The rank of core gut microbiome in Pakistani participants (A) and Chinese participants(B) at genera level, and the different core genera in Chinese and Pakistani participants (C).
